# Assessing aberrant muscle activity patterns via the analysis of surface EMG data collected during a functional evaluation

**DOI:** 10.1186/s12891-018-2350-x

**Published:** 2019-01-05

**Authors:** Fatemeh Noushin Golabchi, Stefano Sapienza, Giacomo Severini, Phil Reaston, Frank Tomecek, Danilo Demarchi, MaryRose Reaston, Paolo Bonato

**Affiliations:** 1 Department of Physical Medicine & Rehabilitation, Harvard Medical School, Spaulding Rehabilitation Hospital, 300 First Ave, Charlestown, MA 02129 USA; 2 School of Electrical and Electronic Engineering, University College Dublin, Dublin, Ireland; 3 Emerge Diagnostics, Carlsbad, CA USA; 4 Oklahoma Spine & Brain Institute, Tulsa, OK USA; 5 Department of Electronics and Telecommunications, Politecnico di Torino, Turin, Italy

**Keywords:** Back pain, Linear regression, Machine learning, Musculoskeletal impairments, Random forest, Surface electromyographic data

## Abstract

**Background:**

Surface electromyographic (EMG) recordings collected during the performance of functional evaluations allow clinicians to assess aberrant patterns of muscle activity associated with musculoskeletal disorders. This assessment is typically achieved via visual inspection of the surface EMG data. This approach is time-consuming and leads to accurate results only when the assessment is carried out by an EMG expert.

**Methods:**

A set of algorithms was developed to automatically evaluate aberrant patterns of muscle activity. EMG recordings collected during the performance of functional evaluations in 62 subjects (22 to 61 years old) were used to develop and characterize the algorithms. Clinical scores were generated via visual inspection by an EMG expert using an ordinal scale capturing the severity of aberrant patterns of muscle activity. The algorithms were used in a case study (i.e. the evaluation of a subject with persistent back pain following instrumented lumbar fusion who underwent lumbar hardware removal) to assess the clinical suitability of the proposed technique.

**Results:**

The EMG-based algorithms produced accurate estimates of the clinical scores. Results were primarily obtained using a linear regression approach. However, when the results were not satisfactory, a regression implementation of a Random Forest was utilized, and the results compared with those obtained using a linear regression approach. The root-mean-square error of the clinical score estimates produced by the algorithms was a small fraction of the ordinal scale used to rate the severity of the aberrant patterns of muscle activity. Regression coefficients and associated 95% confidence intervals showed that the EMG-based estimates fit well the clinical scores generated by the EMG expert. When applied to the clinical case study, the algorithms appeared to capture the characteristics of the muscle activity patterns associated with persistent back pain following instrumented lumbar fusion.

**Conclusions:**

The proposed approach relies on EMG-based measures to generate accurate estimates of the severity of aberrant patterns of muscle activity. The results obtained in the case study suggest that the proposed technique is suitable to derive clinically-relevant information from EMG data collected during functional evaluations.

## Background

Nearly 50% of people living in Western countries experience a musculoskeletal disorder at some point in time during their adult life and the majority of these individuals report related long-term problems [1–3]. Musculoskeletal disorders are typically associated with acute and chronic pain [4, 5]. Low back pain and neck-shoulder pain are among the most common complaints associated with musculoskeletal disorders [6–10]. These conditions are often accompanied by motor dysfunction, i.e. aberrant patterns of muscle activity that lead to non-physiological postures and trajectories of movements as well as non-physiological loads on joints and ligaments [11–14]. The development of clinical intervention plans often relies on observations gathered during the performance of functional evaluations consisting of batteries of motor tasks that mimic activities of daily living (e.g. walking, lifting a box). During these evaluations, clinicians collect biomechanical and electromyographic (EMG) data to assess the severity of motor dysfunction [11–14].

Gathering biomechanical data allows clinicians to identify range of motion limitations, the inability of subjects to produce the desired force output, and aberrant movement patterns [15, 16]. In turn, EMG recordings allow clinicians to detect the root causes of non-physiological postures and movement trajectories such as abnormal readings at rest, muscle hyperactivity, muscle spasms, patterns of compensatory activity of synergistic and/or antagonistic muscles, lack of inhibition of muscle activity, and other aberrant muscle activation patterns [17–23]. Whereas the use of biomechanical measures based on a quantitative analysis of the data is relatively common [15, 16], the analysis of EMG data is typically carried out via visual inspection of the EMG recordings and the use of ordinal scales to rate the severity of abnormally high levels of muscle activity, the presence/absence and severity of muscle spasms, and the observation of non-physiological patterns of recruitment and de-recruitment of the muscles’ motor units as reflected by the amplitude modulation of the surface EMG recordings [24].

The observations gathered by EMG experts vary in complexity according to the task performed by the subject. When subjects are observed in static conditions (e.g. while sitting on a chair or standing still), EMG experts would first assess if the general level of EMG activity is physiological or if it reflects a non-physiological behavior (e.g. muscle hyperactivity) [25, 26]. This observation could be translated into a quantitative measure because the level of EMG activity is associated with the amplitude of the EMG recordings (e.g. the root-mean-square value of the EMG data). A second observation of significant relevance in the analysis of EMG recordings performed in static conditions is the presence/absence and severity of muscle spasms [27, 28]. Muscle spasms are unexpected bursts of muscle activity associated with the recruitment of individual motor units, a pool of motor units that are recruited quasi-periodically in a synchronized manner, or the sustained activity of a pool of motor units [27, 28]. Quantitative measures to capture these behaviors rely on EMG data features associated with the characteristics of the recruitment of motor units during muscle spasms, including data features derived in the time and frequency domains [27, 28].

More complex observations are gathered via visual inspection of the surface EMG data collected in dynamic conditions. EMG experts assess the characteristics of the recruitment and de-recruitment of the muscles’ motor units as reflected by the amplitude modulation of the EMG recordings [29, 30]. They determine the rate of increase and decrease in force production in relation to characteristics of the task performed by the subject such as the range of motion spanned during the task. They identify instances marked by the lack of inhibition of muscle activity (e.g. as in the flexion-relaxation phenomenon [31–33]) that have been associated with musculoskeletal disorders and related pain.

This study aimed to test the hypothesis that clinical scores generated by an EMG expert via visual inspection of the surface EMG recordings collected during a functional evaluation can be accurately estimated via automated analysis of the EMG data using ad hoc algorithms. This is relevant because the analysis of EMG recordings via their visual inspection is time-consuming, a factor that significantly hinders the use of EMG recordings in the clinic. To address this problem, algorithms were developed using an existing database of surface EMG recordings collected during functional evaluations performed using an approach referred to as the Electrodiagnostic Functional Assessment (EFA) [34–36]. The complexity of the algorithms varied according to the complexity of the clinical observations performed via visual inspection of the EMG recordings. The algorithms ranged in complexity from a simple model relying on a single EMG data feature to a set of EMG data features as input to a regression implementation of a Random Forest [37] (i.e. a collection of decision trees).

The algorithms were then applied to EMG data collected to evaluate a subject with persistent back pain after instrumented lumbar fusion who underwent hardware removal [38]. Hardware removal in subjects who have undergone instrumented lumbar fusion and experience back pain is a controversial elective treatment [38–40]. Subjects with recurrent back pain after instrumented lumbar fusion are evaluated by clinical exam, palpation of the lumbar spine, radiographic tests - including lumbar x-rays with flexion/extension views -, post-operative MRI, myelogram CT scan, bone scans, and diagnostic injections consisting of lumbar hardware blocks, selective root blocks, and sacroiliac joint injections. Unfortunately, these diagnostic techniques have been shown to be poor predictors of the outcomes of lumbar hardware removal [38–40]. Very few retrospective studies have been carried out to evaluate the outcomes of lumbar hardware removal in subjects reporting back pain despite an apparent solid fusion and in absence of any obvious pain generator (e.g. persistent neurologic impingement or adjacent level degeneration) [38–41]. Hence, there is a pressing need for identifying reliable predictors of lumbar hardware removal outcomes [42].

## Methods

The data analysis algorithms presented in this manuscript were developed by relying on a database of surface EMG recordings previously collected during functional evaluations performed using an approach referred to as the Electrodiagnostic Functional Assessment (EFA) [34–36]. This section provides a description of the protocols utilized to collect the EMG data and describes the algorithms developed in the study. It also presents the application of the algorithms to a clinical case of lumbar hardware removal in a subject who had previously undergone instrumented lumbar fusion and reported post-operative back pain.

### Surface EMG data

The data had been previously gathered using two EFA-based protocols consisting of a battery of static and dynamic tests designed to assess individuals with cervical pain and back pain, respectively. The dataset included recordings from 62 subjects of age ranging between 22 and 61 years. Fifty-six of these individuals were males. Forty-six of the subjects were evaluated using both protocols. The remaining 16 subjects were evaluated using only one protocol.

For the first protocol, surface EMG recordings were gathered bilaterally from the following muscles of the neck, shoulders, arms, and the thoracic section of the back: scalene, upper trapezius (two channels were recorded using electrodes positioned on the descending muscle fibers below the occipital bone and on the transverse muscle fibers near the acromion, respectively), middle trapezius, longissimus thoracis, middle deltoid, biceps brachii, and triceps. Surface EMG recordings were collected during two static tests, namely 1) while subjects sat still for about 20 s (a condition herein referred to as *rest sitting*) and 2) while they stood without moving for about 20 s (a condition herein referred to as *rest standing*). Surface EMG recordings were also collected during three dynamic tests: 1) flexion/extension, rotation, and lateral movement of the head (herein referred to as *head movements*), 2) shoulder shrug and overhead reaching with both arms (herein referred to as *shoulder and arm movements*), and 3) a lifting functional capacity evaluation (FCE) (i.e. pulling a bar attached to a load-cell with the bar positioned at waist level and the knees fully extended) first with pronation of the forearms and then with supination of the forearms (this condition is herein referred to as *FCE lifting with extended knees*).

For the second protocol, surface EMG data was gathered bilaterally from the following muscles of the lower back and legs: paraspinal at L2, latissimus dorsi, gluteus maximus, biceps femoris, rectus femoris, tibialis anterior, and gastrocnemius (medial head). Surface EMG recordings were collected during the same static tests as per the protocol summarized above (i.e. *rest sitting* and *rest standing*) and during three dynamic tests: 1) flexion/extension, rotation, and lateral movement of the trunk (herein referred to as *trunk movements*), 2) taking two steps, kneeling, and standing up (herein referred to as *walking and kneeling*), and 3) a lifting functional capacity evaluation (i.e. pulling a bar attached to a load-cell with the bar positioned at knee height, the knees in a flexed position, and the arms fully extended) first with pronation of the forearms and then with supination of the forearms (this condition is herein referred to as *FCE lifting with flexed knees*).

Disposable adhesive silver/silver-chloride electrodes were utilized to collect the EMG data. The electrodes were circular in shape (approximately 5 mm in diameter) and had a layer of conductive gel to achieve low skin-electrode impedance. The Surface Electromyography for Non-Invasive Assessment of Muscles (SENIAM) recommendations [43] were followed to position the electrodes on the muscle belly of the selected muscles. The distance between the centers of electrode pairs was approximately 15 mm. Before attaching the electrodes to the skin, the skin was carefully cleaned using alcohol wipes and shaved when necessary. The electrodes were connected to wireless Bluetooth units (Shimmer Research, Dublin Ireland) that transmitted the EMG data to a base station connected to a computer. The wireless units were equipped with a DC-coupled unit (ADS1292R by Texas Instruments, Dallas TX) that provided the analog front-end as well as the analog-to-digital conversion. The data was sampled at 1024 Hz. The data was available prior to initiation of the study as part of a database meant to be used to develop algorithms to facilitate the analysis of data collected during functional evaluations. The database also contained data collected using a load-cell (LSB302 by Futek, Irvine CA) that relied on the above-mentioned wireless Bluetooth units and base station to simultaneously collect EMG and force data when required by the EFA-based protocol (Emerge Diagnostics, Carlsbad CA).

An EMG expert had previously annotated the surface EMG recordings performed during the above-described EFA-based protocols [34–36] using criteria commonly used in a clinical context to capture the level of muscle activity (including its laterality), the presence/absence and severity of muscle spasms, and the muscles’ motor unit recruitment characteristics associated with the EMG amplitude modulation. Clinical scores were generated via visual inspection of the EMG data using an ordinal scale from 0 to 10 designed to capture the magnitude of each phenomenon of interest (e.g., an *activity level* score equal to 0 was associated with recordings marked by very low amplitude of the EMG data, whereas an *activity level* score equal to 10 was associated with recordings marked by very large ampitude of the EMG data). Surface EMG recordings gathered during the static tests were associated with clinical scores for *activity level* and *spasm severity*. Surface EMG recordings gathered during the dynamic tests were associated with clinical scores for *activity level* and *amplitude modulation*. From the clinical scores for *activity level*, the EMG expert also derived *laterality of activity* scores by computing the absolute value of the difference between the scores for the right and the left side of the body for each muscle.

**Fig. 1:**
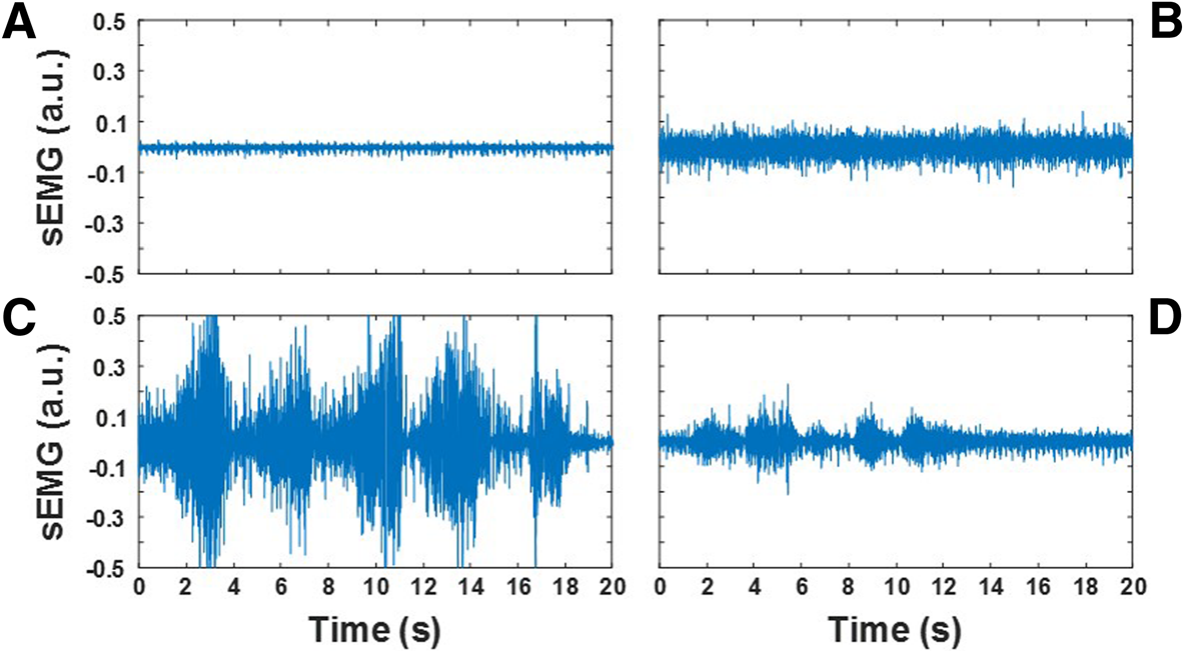
Examples of the surface EMG (sEMG) recordings utilized in the study to develop data analysis techniques suitable to generate estimates of the clinical scores for *activity level* and *spasm severity* for the static tests and for *activity level* and *amplitude modulation* for the dynamic tests. The recordings were collected from the paraspinal muscles at L2. Panels **a** and **b** show EMG data collected during the *rest sitting* test. Panels **c** and **d** show the EMG data collected during the *FCE lifting with flexed knees* test

Figure 1 shows an example of the EMG recordings selected from the dataset utilized in the study. The recordings shown in the figure were collected from the paraspinal muscles at L2 during the *rest sitting* test (Figs. 1a and b) and the *FCE lifting with flexed knees* test (Figs.1c and d). Figures 1a and c show data collected from a subject with very low level of muscle activity (*activity level* = 0) during the *rest sitting* test and high level of EMG activity and amplitude modulation (*activity level* = 10 and *amplitude modulation* = 10) during the *FCE lifting with flexed knees* test. Figures 1b and d show data collected from a subject displaying an elevated level of muscle activity (*activity level* = 5) during the *rest sitting* test and a modest level of activity and minimum amplitude modulation (*activity level* = 3 and *amplitude modulation* = 2) during the *FCE lifting with flexed knees* test.

A low level of muscle activity is expected in physiological conditions during the *rest sitting* test. In contrast, an elevated level of muscle activity is often observed in subjects with back pain. An elevated level of activity of the back muscles has been associated with postural changes that are common acutely in individuals who suffered a back injury and chronically in those who experience chronic back pain [26]. The recordings carried out during the static tests were also rated for *spasm severity*. In individuals with back pain, muscle spasms are believed to be triggered by the activity of nociceptive receptors responding to strain and soft tissue damage [17]. They can be observed in the EMG recordings as short bursts of activity or as sustained activity associated with large action potentials due to the synchronization of motor units [28]. The evaluation of the severity of muscle spasms in the latter case requires close inspection of the EMG recordings to identify the presence of action potentials at quasi-regular time intervals. Using these criteria, the EMG expert rated the recordings in Figs. 1a and b for *spasm severity* as 0 and 5, respectively. A high level of EMG activity and prominent amplitude modulation is expected when healthy subjects perform a functional capacity evaluation. The latter is expected because of the pattern of motor unit recruitment and de-recruitment that marks each burst of EMG activity associated with changes in the force generated by the muscle/s of interest. In contrast, a modest level of activity and minimum amplitude modulation is expected in individuals with back pain who display avoidance patterns [12].

### Data analysis algorithms

Algorithms were developed using the Matlab (The MathWorks, Natick MA) programming environment. Estimates of the clinical scores were derived via the analysis of surface EMG data by filtering the raw EMG recordings, computing the EMG envelope, and extracting data features from the filtered EMG data and from the EMG envelope. Filtering the raw EMG data was achieved using an 8th order elliptic band-pass filter with cut-off frequencies equal to 20 and 400 Hz. This choice of cut-off frequencies allowed us to significantly attenuate low-frequency components associated with motion artifacts as well as high-frequency components associated with the instrumentation noise. The EMG envelope was derived by low-pass filtering the full-wave rectified raw EMG data with a 7th order elliptic filter with cut-off frequency equal to 10 Hz. We empirically determined the optimality of this cut-off frequency as suitable to preserve “high-frequency” amplitude modulation components relevant for an accurate estimation of clinical scores (e.g. *spasm severity* scores). Then different data features were derived to estimate each clinical score. Estimates of the *activity level* scores for the static tests were derived using the root-mean-square (RMS) value of the EMG envelope. This data feature appeared to be suitable to address the problem at hand because we observed that the EMG expert generated the *activity level* scores based on visual inspection of the EMG data amplitude. Estimates of the *spasm severity* scores for the static tests were derived using three data features: the RMS value of the filtered EMG data and the EMG data energy between 50 and 100 Hz and between 100 and 200 Hz. The energy of the EMG data in each of the above-mentioned bandwidths was estimated by deriving the periodogram of the EMG time series (using 1 s rectangular windows with 50% overlap) and integrating the EMG frequency components in the bandwidth of interest. These data features were selected because the EMG expert generated the *spasm severity* scores by considering both the amplitude of the EMG data and its frequency content. The latter was done in an attempt to identify when a prevalent number of type II fibers vs type I fibers were recruited. The frequency bandwidths utilized to derive the above-mentioned parameters were determined empirically. Finally, estimates of the clinical scores for the dynamic tests were derived using the following EMG data features: the RMS value of the EMG envelope, the range spanned by the filtered EMG data (i.e. the peak-to-peak amplitude of the time series), the RMS value of the EMG envelope during the time intervals when the muscle was silent or minimally active, the duration of time during which the muscle was active, the RMS values derived for the three main bursts of EMG activity, the average RMS value of the dominant frequency components of the EMG data, and the variance of the EMG envelope during the three main bursts of activity. The bursts of EMG activity were identified by using a 1 s sliding (by steps of 0.25 s) rectangular window, estimating the RMS value of the EMG envelope within each 1 s time interval, and selecting non-overlapping time intervals associated with the three largest RMS values. The average RMS value of the dominant components of the EMG data was derived by detecting the intervals during which the EMG envelope exceeded 1.5 times the RMS value of the EMG envelope during the entire test. These data features were selected because we observed that the EMG expert generated the clinical scores by considering the amplitude of the EMG data and characteristics of its modulation that appeared to be well captured by the above-mentioned parameters.

Estimates of the *activity level* scores for the static tests were derived by using the RMS value of the EMG envelope as a “proxy” of the clinical scores. This choice was motivated by the observation that the *activity level* scores and the corresponding RMS values of the EMG envelope appeared to be highly correlated. This choice was made based on a qualitative observation of the data and later confirmed by our quantitative analyses (see the *Results* section). *Activity laterality* scores were estimated by taking the absolute value of the difference between the *activity level* scores derived from the EMG recordings for the right and the left side of the body for a given muscle. The *spasm severity* scores were estimated by using a linear regression model relying on the above-mentioned EMG data features as independent variables. Finally, a linear regression model as well as a regression implementation of a Random Forest with the above-listed EMG data features as input variables were used to estimate the *activity level* scores and the *amplitude modulation* scores for the dynamic tests. A linear and a non-linear model for the analysis of the EMG data collected during the dynamic tests were implemented and compared because preliminary inspection of the EMG data features suggested that the output variables (i.e. the clinical scores) and the input variables were non-linearly related. The estimates of the *activity level* scores were also used to derive estimates of the *activity laterality* scores as described above for the static tests. Separate models were developed for the EMG data collected during each test.

The derivation of the linear regression coefficients for the static tests took into consideration the unbalance across classes corresponding to the different values of the clinical scores. This is a standard technique utilized in machine learning to avoid fitting well the data pertaining to classes with a large number of samples and fitting poorly the data pertaining to classes with a small number of samples [44]. Accordingly, because the instances of *spasm severity* scores equal to 0 were significantly larger than the instances for other score values, we randomly down-sampled to 10% of the total sample size the class associated with clinical scores equal to 0. We did so 10 times and hence derived 10 datasets to fit the model (i.e. 10 training sets). Also, because a relatively small number of instances with *spasm severity* scores greater than 5 was available, first, a model was developed using the instances with scores ranging from 0 to 5. Then, the model was applied to all the data. This process was repeated 10 times, for each of the datasets derived as mentioned above. Finally, the coefficient values of the model obtained for each of the 10 datasets were averaged. This approach was also used to derive linear regression models for the dynamic tests. The score distribution across classes for the dynamic tests was different than the score distribution for the static tests. The procedure was modified accordingly by down-sampling the class with the largest number of instances. The importance of each independent variable to generate the clinical score estimates was assessed based on the magnitude of the model’s standardized coefficients.

The Random Forest [37] models were derived using bootstrap aggregation (i.e. bagging [45]) of 100 regression trees with a minimum leaf size of 10. This method is very robust even when dealing with unbalanced classes. Hence, it was not necessary to rebalance the classes. A 10-fold cross-validation was used to derive the clinical score estimates. The importance of the input variables (i.e. the EMG data features) was assessed by measuring the increase in prediction error when the values of the input variables were permuted across the out-of-bag observations. This is a well-established method to assess the relevance of the input variables of a Random Forest [46].

The accuracy of the estimates derived using the above-described methods was assessed by deriving the root-mean-square error (RMSE) for each model. In addition, a linear regression between the clinical scores generated by the EMG expert and those estimated by the above-described EMG-based algorithms was computed for each clinical score. Regression coefficients as well as 95% confidence intervals were computed for each linear regression.

### Clinical case study

A subject with persistent back pain after instrumented lumbar fusion was evaluated using an EFA-based protocol prior to and after lumbar hardware removal. Before the lumbar hardware removal surgery, the subject reported low back pain with a severity level of 5 out of 10 on a visual-analog scale. The pain radiated to the left leg and was of higher intensity when stepping down with the left leg. Numbness and tingling were reported around the lumbar incision. The pain was clinically managed with medications. Inspection of the lumbar hardware during the surgery made apparent that one of the screws had migrated in the spinal canal and was likely to be responsible for the symptoms reported by the subject. The lumbar hardware removal led to a significant decrease in back pain, a decrease in pain medications, and an improvement in the subject’s functional ability.

The study was approved by the Western Institutional Review Board. The subject was evaluated using a modified version of the above-described protocol for the assessment of subjects with back pain. The battery of tests used to assess this subject included the following: 1) a series of *rest sitting* and a *rest standing* tests at the beginning of the evaluation; 2) a *trunk flexion/extension movement* test, which consisted of bending forward, returning to the upright position, bending backward, and returning to the upright position; 3) a *trunk lateral movement* test, which consisted of bending laterally first to the right and then to the left; 4) a *walking and kneeling* test; 5) an additional set of *rest standing* tests; 5) a series of *FCE lifting with flexed knees* tests; and 6) a final set of *rest standing* and *rest sitting* tests. During the evaluation, surface EMG data was collected bilaterally from the following muscles: paraspinals at L2 and L5, latissimus dorsi, gluteus maximus, biceps femoris, and rectus femoris. In addition, triaxial accelerometer recordings were gathered using wireless sensor units positioned at L1 and S1 to derive range of motion data. Also, a load-cell was used during the FCE tests.

## Results

The results of the analyses carried out using the above-described techniques are presented in the following, first for the surface EMG data collected during the performance of the static tests and then for the data collected during the performance of the dynamic tests. The datasets utilized in this part of the study were part of the above-mentioned database of EMG recordings collected from 62 subjects. In addition, this section of the manuscript summarizes the results obtained by using the algorithms to surface EMG recordings collected as part of the above-described clinical case study.

### Analysis of the EMG data collected during the static tests

**Fig. 2:**
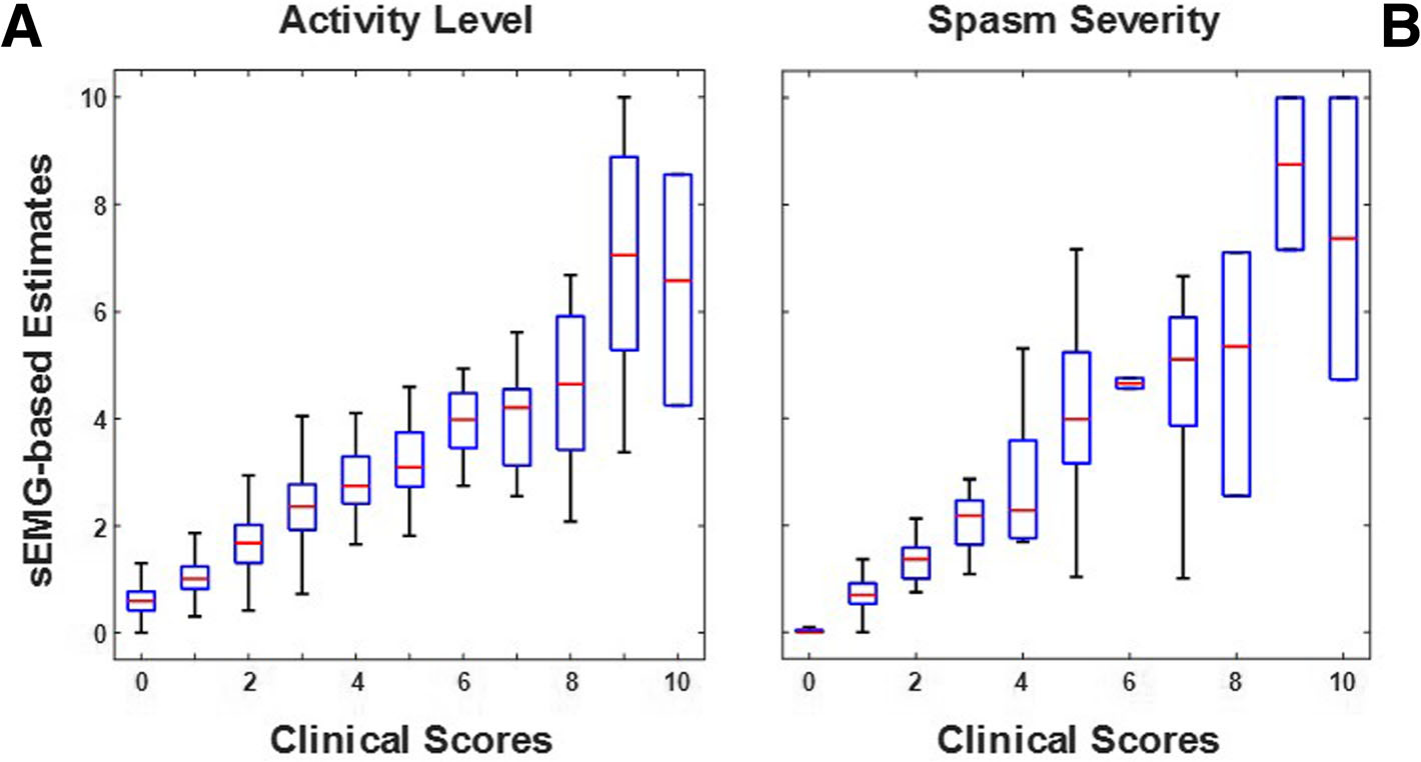
Box plots of the surface EMG (sEMG)-based estimates vs. the clinical scores generated by the EMG expert for the static tests. Panel **a** - Estimates of the *activity level* scores. Panel **b** - Estimates of the *spasm severity* scores

Figure 2 shows a box plot representation of the results obtained using the above-described algorithms for the analysis of data collected during the performance of static tests. The plots show aggregate results for the *rest sitting* and the *rest standing* tests. For each class (i.e. for each clinical score value), the plots display the median value of the EMG-based estimates (red horizonal line), a box spanning the interquartile range of the distribution of the clinical score estimates, and whiskers that span the range from the minimum to the maximum estimated clinical score values. It is worth noticing that outliers (defined as values exceeding the first and third quartile more than 1.5 times the interquartile range) are not shown in Fig. 2. However, outliers were not removed when assessing the accuracy of the EMG-based estimates (i.e. when estimating the RMSE values as well as the regression coefficients and corresponding 95% confidence intervals). It is also worth noticing that when the number of instances in a class is small, the interquartile range and the range between the minimum and the maximum estimated clinical score values might be undistinguishable. Hence, the whiskers might be not clearly displayed in the box plot representation. This is the case for a few classes in Fig. 2.

Figure 2a shows the surface EMG-based estimates of the clinical scores for *activity level*. The estimates were derived using the RMS value of the EMG envelope as a proxy for the *activity level* scores. Although the plot shows aggregate results across testing conditions, separate models were derived for the *rest sitting* and the *rest standing* tests. The regression coefficients and corresponding 95% confidence intervals derived from the data collected during the *rest sitting* and the *rest standing* tests were equal to 0.89 ± 0.04 (regression coefficient ± 95% confidence interval value) and 1.19 ± 0.04, respectively. The small 95% confidence interval values associated with the regression coefficients for both the *rest sitting* and the *rest standing* scores are indicative of a good correlation between the EMG-based estimates and the clinical scores generated by the EMG expert.

Figure 2b shows the surface EMG-based estimates of the clinical scores for *spasm severity*. The estimates were derived using a linear regression model with the following three independent variables as input: the RMS value of the filtered raw EMG data and the EMG data energy between 50 and 100 Hz and between 100 and 200 Hz. Figure 2b shows aggregate results for the *rest sitting* and the *rest standing* tests. Regression coefficients and associated 95% confidence intervals were derived separately for the *rest sitting* (0.63 ± 0.02) and the *rest standing* (0.71 ± 0.02) tests. Both models used the above-mentioned independent variables as input variables. The standardized coefficients and corresponding 95% confidence intervals for the *rest sitting* test were 0.02 ± 0.07, 0.58 ± 0.05, and − 0.03 ± 0.06. The standardized coefficients and corresponding 95% confidence intervals for the *rest standing* test were 0.08 ± 0.11, 0.42 ± 0.07, and 0.14 ± 0.06. The values of the standardized coefficients suggest that the EMG data energy between 50 and 100 Hz is the most relevant independent variable to estimate the *spasm severity* scores.

Table 1 provides a summary of the above-discussed results. The table shows the RMSE values as well as the regression coefficients and associated 95% confidence interval values for the EMG-based estimates of the clinical scores for *activity level* and for *spasm severity* derived for the static tests. The table shows also the results for the *laterality of activity* scores. The RMSE values range from 0.37 (for the estimation of the *spasm severity* scores for the *rest sitting* test) to 0.98 (for the estimation of the *laterality of activity* scores for the *rest standing* test). The regression coefficient values range from 0.63 to 1.19 and the associated 95% confidence intervals range from 0.02 to 0.06. These results indicate that the proposed methods allow one to achieve accurate estimates of the clinical scores for *activity level*, *spasm severity* and *laterality of activity* scores.

**Table 1:** RMSE, regression coefficients (RC) and associated 95% confidence intervals (CI) of the surface EMG-based estimates of the clinical scores for the static tests

	Activity Level		Spasm Severity		Laterality of Activity	
	RMSE	RC ± CI	RMSE	RC ± CI	RMSE	RC ± CI
rest sitting	0.72	0.89 ± 0.04	0.37	0.63 ± 0.02	0.75	0.88 ± 0.05
rest standing	0.86	1.19 ± 0.04	0.43	0.71 ± 0.02	0.98	0.84 ± 0.06

### Analysis of the EMG data collected during the dynamic tests

**Fig. 3:**
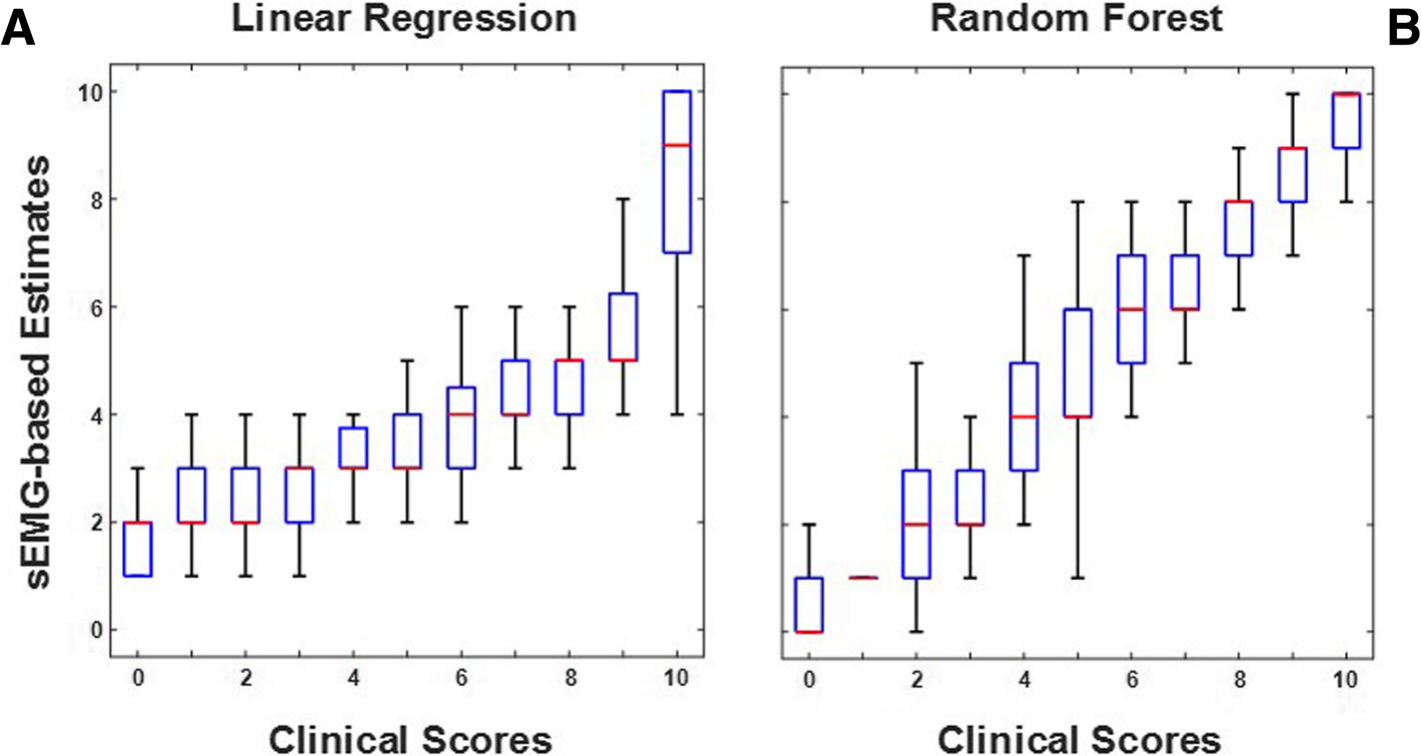
Box plots of the surface EMG (sEMG)-based estimates of the *amplitude modulation* scores vs. the corresponding clinical scores for the *FCE lifting with flexed knees* test. Panel **a** - Estimates obtained using a linear regression model. Panel **b** - Estimates obtained using a Random Forest-based algorithm

Figure 3 shows an example of the surface EMG-based estimates of the clinical scores for the dynamic tests. Specifically, the figure shows the estimated *amplitude modulation* scores using a linear regression model (Fig. 3a) and using a Random Forest-based algorithm (Fig. 3b) vs. the scores generated by the EMG expert for the *FCE lifting with flexed knees* test. The box plot representation of the surface EMG-based score estimates shows that the Random Forest-based algorithm generated more accurate estimates than the linear regression model. This qualitative observation is confirmed by the estimation errors associated with these two different approaches to achieve the clinical score estimates. In fact, the RMSE of the surface EMG-based estimates shown in Fig. 3a (for the linear regression model) was 2.09 whereas the RMSE of the estimates shown in Fig. 3b (for the Random Forest-based model) was 1.74.

Tables 2 and 3 show a summary of the RMSE values as well as the regression coefficients and associated 95% confidence interval values for all the dynamic tests. Table 2 shows the values for the surface EMG-based estimates derived using linear regression models. Table 3 shows the values for the surface EMG-based estimates derived using Random Forest-based models. The tables show the RMSE values as well as the regression coefficients and associated 95% confidence interval values for all the test conditions for *activity level*, *amplitude modulation*, and *laterality of activity*. The RMSE values obtained using the Random Forest-based models were on average smaller than those obtained using the linear regression models with improvements approaching 50% for the *FCE lifting with extended knees* test. The regression coefficients and 95% confidence intervals values show a similar trend. The regression coefficients obtained using the linear regression models ranged from 0.84 to 1.72 with 95% confidence interval values ranging from 0.01 to 0.18. The regression coefficients obtained using the Random Forest-based models ranged from 0.88 to 1.04 with 95% confidence interval values ranging from 0.02 to 0.08. Overall, these results indicate that Random Forest-based models are more suitable than linear regression models to estimate *activity level*, *amplitude modulation*, and *laterality of activity* scores. Analysis of the relevance of the EMG data features used as input to the Random Forest-based models showed similar results for the *activity level* and *amplitude modulation* scores. In both cases, the most relevant input variables to estimate the clinical scores were the RMS values derived for the three main bursts of EMG activity, and the variance of the EMG envelope computed for the largest burst of muscle activity.

**Table 2:** RMSE values, regression coefficients (RC) and associated 95% confidence intervals (CI) of the surface EMG-based estimates of the clinical scores for the dynamic tests derived using a linear regression model

	Activity Level		Amplitude Modulation		Laterality of Activity	
	RMSE	RC ± CI	RMSE	RC ± CI	RMSE	RC ± CI
head movements	1.13	1.31 ± 0.02	1.05	1.28 ± 0.03	1.43	1.21 ± 0.01
shoulder and arm movements	1.96	1.06 ± 0.01	2.16	1.21 ± 0.01	1.76	1.02 ± 0.05
FCE lifting with extended knees	1.97	1.01 ± 0.13	2.31	1.17 ± 0.01	1.88	0.75 ± 0.08
trunk movements	1.20	1.19 ± 0.18	1.43	1.37 ± 0.02	1.41	1.07 ± 0.08
walking and kneeling	1.87	1.04 ± 0.02	2.00	1.32 ± 0.04	1.72	0.86 ± 0.06
FCE lifting with flexed knees	1.85	1.03 ± 0.02	2.09	1.72 ± 0.04	1.85	0.84 ± 0.08

**Table 3:** RMSE, regression coefficients (RC) and associated 95% confidence intervals (CI) of the surface EMG-based estimates of the clinical scores for the dynamic tests derived using a regression implementation of a Random Forest

	Activity Level		Amplitude Modulation		Laterality of Activity	
	RMSE	RC ± CI	RMSE	RC ± CI	RMSE	RC ± CI
head movements	1.07	0.98 ± 0.04	1.04	1.01 ± 0.07	1.46	0.88 ± 0.08
shoulder and arm movements	1.13	1.00 ± 0.03	1.45	1.00 ± 0.03	1.41	0.93 ± 0.05
FCE lifting with extended knees	1.12	0.98 ± 0.02	1.22	0.98 ± 0.02	1.37	0.93 ± 0.06
trunk movements	1.17	1.00 ± 0.04	1.30	1.04 ± 0.05	1.34	0.98 ± 0.05
walking and kneeling	1.25	1.01 ± 0.03	1.64	1.01 ± 0.05	1.36	0.96 ± 0.06
FCE lifting with flexed knees	1.36	1.00 ± 0.03	1.74	1.00 ± 0.04	1.52	1.01 ± 0.06

**Fig. 4:**
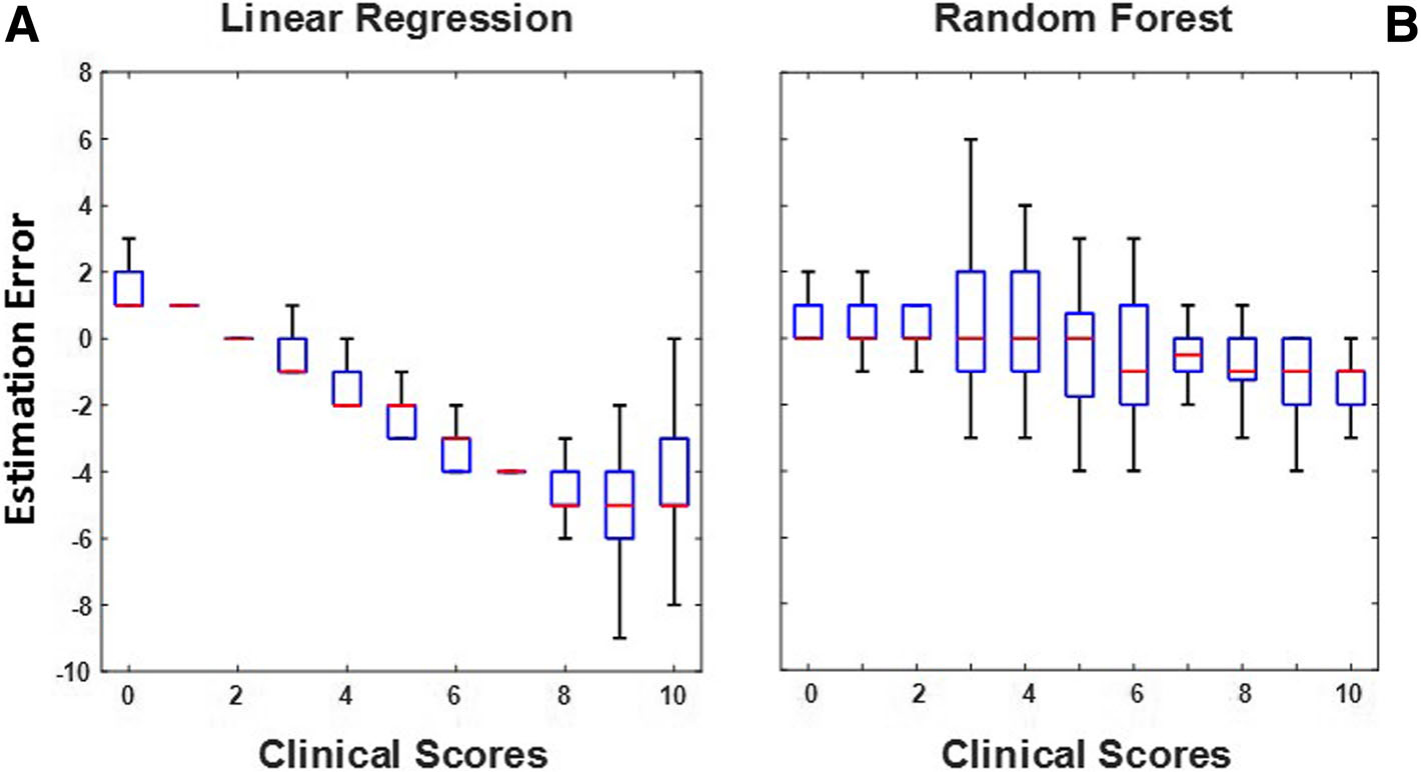
Errors associated with the EMG-based estimates of clinical scores derived using a linear regression model (panel **a**) and using a regression implementation of a Random Forest-based model (panel **b**). The plots show data for the *amplitude modulation* scores derived from EMG recordings collected during the *FCE lifting with extended knees* test

The improved accuracy of the EMG-based estimates obtained using Random Forest-based models over those obtained using linear regression models is apparent when one examines plots of the estimation errors as a function of the clinical scores generated by the EMG expert. An example of such plots is shown in Fig. 4. The plots in Fig. 4 allow one to compare the results obtained with the above-mentioned approaches when estimating the *amplitude modulation* scores for the *FCE lifting with extended knees* test. The plot on the left side (panel a) shows a clear trend in the bias affecting the EMG-based estimates. This is likely to be the case because the linear regression model fails to account for the non-linearity of the relationship between the input variables and the *amplitude modulation* scores. Such relationship is instead properly captured by the model based on a regression implementation of a Random Forest. In fact, the results show a modest trend in estimation bias and a relatively constant variance of the EMG-based estimates across clinical scores (panel b). Similar plots were obtained for all the EMG-based estimates for all the dynamic tests. Inspection of the plots showed similar differences as those shown in Fig. 4 between the estimates derived using a linear regression model and the estimates derived using a Random Forest-based algorithm. These observations indicate that Random Forest-based algorithms are more suitable than linear regression models to estimate clinical scores for recordings collected during dynamic tests.

### Clinical application of the algorithms

The algorithms developed in the study were applied to a dataset collected from a subject with persistent back pain after instrumented lumbar fusion who underwent hardware removal. The subject underwent functional evaluations prior to and after lumbar hardware removal using the protocol and equipment described in the *Methods* section.

**Fig. 5:**
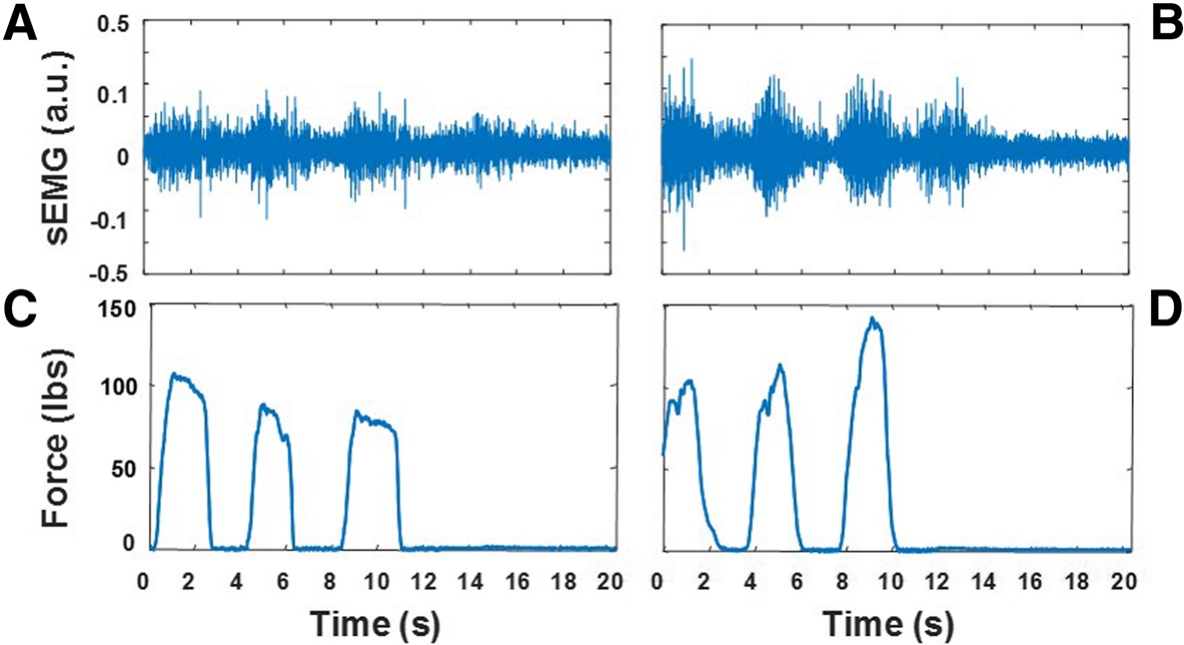
Surface EMG (sEMG) recordings from the left paraspinal muscle at L2 and load-cell data collected during the performance of the *FCE lifting with flexed knees* test. Panels **a** and **c** show data collected before the hardware removal surgery. Panels **b** and **d** show data collected after the surgery. The EMG recording before the lumbar hardware removal surgery shows a lower level of activity and a more modest amplitude modulation compared to the data collected after surgery. It is worth noticing that Panels **a** and **b** show three bursts of EMG activity associated with the lifting task and a fourth burst of activity associated with returning to the upright position

Figure 5 shows an example of the surface EMG and load-cell data collected during the study. The panels of Fig. 5 show the EMG recordings from the left paraspinal muscle at L2 and the load-cell data collected during the performance of the *FCE lifting with flexed knees* test prior to (panels a and c) and after (panel b and d) the hardware removal surgery. The *activity level* and the *amplitude modulation* scores prior to the hardware removal surgery were 2 and 0, respectively. The corresponding clinical scores after the hardware removal surgery were 4 and 3, respectively. These clinical scores captured an increase in the EMG level of activity and its amplitude modulation. In fact, the RMS value of the bursts of EMG activity after the hardware removal surgery was approximately 40% larger than those detected before the surgery. The load-cell data also showed a marked increase in force output following the lumbar hardware removal surgery. In fact, the load-cell output barely reached 100 lbs before the surgery whereas it approached 150 lbs after the surgery. It is worth noticing that the improvements in the patterns of muscle activity shown in Fig. 5 were observed at the level of the lumbar fusion.

Additional observations of interest were gathered during the other tests performed as part of the EFA-based protocol that was used to clinically assess the subject. During the *rest standing* test at the beginning of the battery of tests, the analysis of the data identified a large level of activity in the right biceps femoris that decreased after the lumbar hardware removal surgery. Spasms were observed in the recordings from the left biceps femoris before surgery that were not present after surgery and compensatory activity in the left rectus femoris that was not present after surgery. The analysis of the surface EMG data collected during the *trunk flexion/extension movement* tests showed an improvement in the amplitude modulation of the data collected from the paraspinal muscles at L2 and at L5, and the gluteus maximus. The data collected during the *trunk lateral movement* tests showed improvements in the patterns of activity of the right and left latissimus dorsi muscles, the paraspinal muscles at L2 and at L5, and the biceps femoris. The data collected during the *walking and kneeling* tests showed bilateral improvements post-surgery in the patterns of activity of the paraspinal muscles at L2 and at L5. Improvements post-surgery were also observed bilaterally in the activity of the paraspinal muscles at L2 and at L5 during the *rest standing* tests that followed the *walking and kneeling* tests and in the activity of the biceps femoris and rectus femoris during the *rest standing* tests that followed the *FCE lifting with flexed knees* tests. No differences were observed during the *rest sitting* tests at the end of the battery of tests.

These observations are clinically relevant. The results of the above-summarized analyses indicate that the subject displayed patterns of muscle activity post-surgery that were closer than the ones observed before surgery to the physiological patterns of activity. These EMG-based observations were associated with improvements in range of motion, force output and in the general status of the individual (e.g. self-report of pain severity). Most importantly, close inspection of the hardware during the surgery showed that one of the screws had migrated in the spinal canal and was likely causing the symptoms reported by the subject. These results should be looked upon as a preliminary example of potential application of the procedures developed in the study. The validity of the proposed approach will have to be confirmed in future clinical studies. Nonetheless, the results herein reported suggest that our approach could be utilized in clinical assessments where other techniques have failed to capture the impact of soft tissue damage causing back pain.

## Discussion

The results presented in this manuscript show that the clinical scores that EMG experts generate via visual inspection of the surface EMG recordings collected during the performance of functional evaluations can be accurately estimated by means of linear and non-linear algorithms that use EMG data features as inputs. The results show that clinical scores for *activity level* and *spasm severity* for static tests can be accurately estimated using linear algorithms. The use of such algorithms leads to RMSE values smaller than 1 point of the 10-point ordinal scale used by the EMG expert to generate the clinical scores. Clinical scores for *activity level* and *amplitude modulation* associated with dynamic tests can be accurately estimated using Random Forest-based algorithms. The estimates generated using such algorithms are marked by a RMSE smaller than 2 points of the 10-point ordinal scale used to generate the clinical scores.

Accurate estimates of the *activity level* scores associated with the static tests were obtained by using the RMS value of the EMG envelope as a proxy for the clinical score. Using such a simple approach was possible because the *activity level* scores and the RMS values of the associated EMG envelope time series showed a clear linear relationship as measured by the linear regression coefficient and 95% confidence interval values shown in Table 1. Accurate estimates of the *spasm severity* scores for the static tests were achieved by using three EMG-based data features as input to a linear regression model: the RMS value of the filtered raw EMG data and the EMG data energy between 50 and 100 Hz and between 100 and 200 Hz. The accuracy of the EMG-based estimates was shown by the low RMSE values and the small regression coefficients and associated 95% confidence intervals associated with the estimates. The model’s standardized coefficients showed the EMG data energy between 50 and 100 Hz to be the most relevant independent variable to estimate the clinical scores. This observation suggests that spasms have a greater impact on the frequency content of the EMG data in the 50–100 Hz bandwidth than on other frequency components of the EMG data. In addition, one would anticipate that the 50–100 Hz bandwidth would be generally marked by a higher signal-to-noise ratio than other portions of the frequency content of the EMG data. This is because noise components are expected to be quasi-uniformly distributed across the entire frequency range, whereas the majority of the EMG frequency components are expected to be in the 50–100 Hz range.

The clinical scores associated with the EMG recordings collected during dynamic tests (i.e. the *activity level* and the *amplitude modulation* scores) were estimated by using linear regression models as well as non-linear models based on a regression implementation of Random Forests [37] (i.e. ensembles of regression trees). A set of EMG-based features was used as input to these models: the RMS value of the EMG envelope, the range spanned by the filtered raw EMG data, the RMS value of the EMG envelope for the time intervals when the muscle was silent or minimally active, the duration of time during which the muscle was active, the RMS values derived for the three main bursts of EMG activity, the average RMS value of the dominant components of the EMG data, and the variance of the EMG envelope during the three main bursts of activity. The non-linear models were found to be more suitable than the linear ones to estimate the *activity level* and the *amplitude modulation* scores for data collected during the dynamic tests. This was anticipated because preliminary inspection of the relationship between the individual input variables and the clinical scores showed a non-linear characteristic. Analysis of the relevance of the EMG data features used to generate the clinical scores using the Random Forest-based models showed that the RMS values derived for the three main bursts of EMG activity, and the variance of the EMG envelope during the largest burst of muscle activity were the most relevant input variables to estimate the clinical scores. This was not unexpected because EMG experts generate the clinical scores for the dynamic tests by primarily observing the characteristics of the EMG amplitude modulation that marks the main bursts of muscle activity associated with the performance of the task of interest.

It is interesting to notice that simpler models were found to be satisfactory to estimate clinical scores for static tests compared to dynamic tests. This could be due in part to the fact that a simpler relationship exists between EMG characteristics and force output during static tests compared to dynamic tests. In fact, during static tests (i.e. isometric muscle contractions), the amplitude of the EMG data is linearly related to the force generated by relevant muscles. Such relationship is more complex during dynamic contractions when additional factors - such as the relative displacement of the surface electrodes with respect to the active muscle fibers - affect the amplitude of the surface EMG data. Furthermore, the analysis of EMG recordings collected during dynamic tests requires consideration of the relationship between the EMG data and the characteristics of the task (e.g. the requirements related to the biomechanics of movement). Hence, it is not surprising that a larger set of EMG-based data features and more complex models (including non-linear models) are needed to accurately estimate clinical scores for dynamic tests than those needed to estimate clinical scores for static tests.

The use of algorithms to automatically generate clinical scores from the EMG data is an interesting alternative to the generation of clinical scores based on visual inspection of the recordings. A first important reason to choose an EMG data feature-based approach is the fact that functional evaluations require the collection of a significant amount of EMG data during the performance of several motor tasks. Hence, visual inspection of the data requires a substantial amount of time. A second reason to choose an EMG data feature-based approach is the fact that the generation of clinical scores via visual inspection of the EMG recordings, although guided by well-established criteria, can be accurately performed only by EMG experts. The EMG-based approach presented in this manuscript is an objective and accurate alternative to the generation of clinical scores via visual inspection of the EMG recordings. In addition, it is worth noticing that EMG-based estimates can be more easily characterized - in terms of their reliability and validity - than clinical scores generated by EMG experts. In fact, the latter are a function of the level of expertise of the individual performing the assessment, whereas the former are generated automatically based on the EMG recordings.

It is worth emphasizing that the method developed in this study is conceptually independent on the specific approach chosen to normalize the EMG data. The normalization of the EMG data has been the subject of numerous studies. In studies with focus on healthy individuals, it is appropriate and relatively easy to normalize the EMG data by the amplitude of the recordings performed during a maximum voluntary contraction. However, in studies with focus on individuals with motor impairments and individuals who experience pain, alternative ways to normalize the EMG data have been proposed because these individuals are not capable of performing a reliable maximum voluntary contraction [47–49]. The methodology herein proposed can be applied to the analysis of EMG data irrespective of the adopted normalization technique as long as the models’ parameters are adjusted accordingly.

Our method complement approaches developed in previous studies with focus on specific EMG data features in the time and the frequency domain [50, 51]. For instance, a significant body of work has been devoted to estimating localized muscle fatigue using traditional spectral analysis techniques for isometric contractions [52–54] and time-frequency analysis techniques for dynamic contractions [53, 55–59]. Contrary to previously proposed techniques that have been focused on capturing specific characteristics of the EMG recordings, the algorithms developed in this study are suitable to capture all the complex aspects of the EMG recordings that are captured via visual inspection of the EMG data by EMG experts. In contrast, other techniques (e.g. the analysis of the frequency content of the EMG data) can capture data features that are not easily identifiable via visual inspection of the data in the time domain.

Furthermore, whereas the algorithms herein presented generate accurate scores that quantify different aspects of the surface EMG recordings, such scores should be interpreted by EMG experts in the context of the evaluation protocol utilized in the study. For instance, spasms have been observed in healthy subjects when lifting large weights [60] and hence a *spasm severity* score greater than zero could be expected in these experimental conditions even in absence of a specific pathology.

The clinical relevance of the proposed technique was highlighted by the clinical case study presented in the manuscript. An individual with persistent back pain after instrumented lumbar fusion who underwent hardware removal was assessed using an EFA-based protocol. The EMG recordings collected during the evaluation were analyzed using the algorithms developed in the study. The algorithms were found to be sensitive to changes in the EMG data and the clinical status of the individual pre- vs post-surgery. Diagnostic methods that are currently used in the clinic have been shown to very often fail in predicting the outcomes of lumbar hardware removal. The results reported in this manuscript show that the proposed approach has significant potential for clinical application. The method should be further assessed in candidates for lumbar hardware removal in absence of any obvious pain generator. This is important because the proposed EMG analysis-based technique appears to have potential for improving the ability of clinicians to predict the outcomes of lumbar hardware removal.

Whereas the EMG recordings used in the study were collected during the performance of specific tasks (i.e. tests that are part of EFA-based protocols), the conceptual development of the algorithms herein proposed is applicable to recordings gathered during the performance of activities of daily living. Furthermore, the technology used to collect the data utilized in the study consists of a set of wearable wireless units that could be used to monitor individuals in the home and community setting. Collecting data outside of the clinic (i.e. in real-life conditions) is of great interest in rehabilitation medicine [61, 62]. Future studies should be focused on evaluating the use of the algorithms herein presented to analyze data collected in unconstrained conditions outside of the clinic.

## Conclusions

The results of this study demonstrate that clinical scores that capture the level of muscle activity, the presence/absence and severity of spasms, and the characteristics of the EMG amplitude modulation can be estimated using linear and non-linear algorithms relying on EMG data features as their inputs. The complexity of these algorithms varies with the complexity of the clinical observations. The clinical case study presented in this manuscript suggests that the proposed algorithms have potential for augmenting the ability of clinicians to predict the outcomes of lumbar hardware removal in individuals who report persistent back pain after instrumented lumbar fusion despite an apparent solid fusion and in the absence of any obvious pain generator. The approach utilized in the study to design EMG-based algorithms is applicable to data collected under unconstrained conditions and hence is potentially suitable to perform clinical evaluations using wearable sensor technology in the home and community settings.

## Abbreviations

CI: Confidence interval
DC: Direct current
EFA: Electrodiagnostic functional assessment
EMG: Electromyographic
FCE: Functional capacity evaluation
RC: Regression coefficient
RMS: Root mean square
RMSE: Root mean square error
sEMG: Surface electromyographic
SENIAM: Surface electromyography for non-invasive assessment of muscles
